# Antioxidant-Rich Polyfloral Bee Pollen Exerts Antimicrobial Activity and Anti-Inflammatory Effect in A549 Lung Epithelial Cells by Modulating the NF-κB Pathway

**DOI:** 10.3390/foods14050802

**Published:** 2025-02-26

**Authors:** Andrea Cavallero, Francesca Vidotto, Cristiana Sbrana, Laryssa Peres Fabbri, Giulio Petroni, Morena Gabriele

**Affiliations:** 1 Institute of Agricultural Biology and Biotechnology, National Research Council, Via Moruzzi 1, 56124 Pisa, Italy; andrea.cavallero@ibba.cnr.it (A.C.); cristiana.sbrana@cnr.it (C.S.);; 2 Department of Biology, University of Pisa, Via Luca Ghini 13, 56123 Pisa, Italy; giulio.petroni@unipi.it

**Keywords:** bee pollen, polyphenols, antioxidant activity, antimicrobial potential, lung inflammation, anti-inflammatory properties, NF-kB pathway

## Abstract

Bee pollen is produced by honeybees from the agglutination of pollen grains belonging to one or more plant species. Although it is intended to be a nutritional source for the hive, its remarkable concentration of nutrients and phytochemicals, combined with its pleasant organoleptic qualities, makes it appealing for human consumption. This study examined the phytochemical content and the antioxidant and antimicrobial activities of a polyfloral bee pollen collected in Tuscany (Italy). Additionally, its less studied anti-inflammatory potential towards tumor necrosis factor-alpha (TNF-α)-inflamed A549 cells was evaluated to assess its possible use in inflammatory respiratory diseases. Bee pollen extract (BPE) was chemically characterized in terms of total polyphenol (20.2 ± 1.5 mg gallic acid equivalents/g fw), flavonoid (9.22 ± 0.64 mg quercetin equivalents/g fw), and carotenoid (10.4 ± 1.4 µg carotenoids/g fw) contents. BPE exerted good antioxidant and antiradical activities in ferric reducing antioxidant power (38.6 ± 4.5 mg Fe^2+^/g fw), oxygen radical absorbance capacity (433.77 ± 18.95 μmol TE/g fw), and 2,2-diphenyl-1-picrylhydrazyl (EC_50_ = 613.8 ± 79.2 μg/mL) assays. Additionally, BPE inhibited the growth of *Bacillus subtilis* and *Pseudomonas stutzeri* (MIC = 10 mg/mL) in the microdilution assay. When TNF-α-inflamed A549 cells were pretreated with BPE (10 and 50 µg/mL), the upregulated interleukin-8 gene and cyclooxygenase-2 gene and protein expression were significantly attenuated. BPE modulated the nuclear factor-κB signaling pathway by decreasing its active phosphorylated form levels. These encouraging results confirm that honeybee pollen is a valuable health-promoting food that could alleviate the inflammatory component of various chronic pulmonary conditions.

## 1. Introduction

Honeybees (*Apis mellifera*, Linnaeus, 1758) are extraordinary social insects that can generate several products of human interest, including honey, bee pollen, propolis, royal jelly, bee bread, and beeswax [1]. Bee pollen is regarded as a complete and nutritious food, and its therapeutic properties were appreciated in ancient Greek, Chinese, and Egyptian folk medicine and the Bible [2,3].

Bee pollen results from the agglutination of flower pollen grains harvested from various insect-pollinated plant species, with honeybee-secreted salivary enzymes and nectar, to form compact pollen loads, sizing from about 1.4 to 4 mm, which are carried to the hive stuck to bees’ rear legs [1,2,3]. Bee pollen is classified as monofloral when at least 45% of the pollen grains share the same taxonomic origin, and as polyfloral when derived from various plant sources, with none being significantly represented [4]. Although the chemical composition of bee pollen is highly varied due to geographical region, climate and season, plant botany, soil type, and bee species, its main constituents are carbohydrates, followed by proteins, free amino acids, and lipids [3,5,6].

The nutritional interest of bee pollen is also linked to the micronutrients it provides, such as fat-soluble vitamins (pro-vitamin A, vitamins D and E), water-soluble ones (vitamins B1, B2, B6, B9, and C), and several minerals in good concentrations [1,3,6,7]. However, it is mainly the richness in secondary metabolites that explains the broad spectrum of biological activities attributed to this product of the hive, namely, antioxidative, anti-inflammatory, antidiabetic, anticarcinogenic, hepatoprotective, nephroprotective, skin-protective, antiallergic, and antimicrobial [3,5,6,8,9,10,11,12]. Indeed, the principal phytochemicals found in bee pollen are carotenoids, phenolic acids, and flavonoid aglycones and glycosides, which are renewed antioxidant health-promoting compounds as well as responsible for pollen’s organoleptic qualities [6,9,13].

Due to these attributes and its affordable market price, bee pollen is considered a highly valuable nutraceutical product and pharmaceutical ingredient, reasons that have granted it recognition as medicine in Germany and that justify further investigation into its beneficial properties [3]. For instance, bee pollen’s capacity to tackle oxidative stress and inflammation, which are recognized key physio-pathological processes underlying human disease, could be a strategy to alleviate chronic inflammatory respiratory diseases such as asthma, chronic obstructive pulmonary disease (COPD), and pulmonary fibrosis. These common diseases lead to impaired air flow through respiratory systems due to chronic inflammation that can disrupt airway tissues’ homeostasis and structure, severely impacting patients’ lives. Despite having their peculiarities in terms of etiology, inflammation localization, degree of fibrosis, and cause of airway obstruction, all of them result from or are exacerbated by pro-oxidative and pro-inflammatory stimuli, primarily cigarette smoking and wood fire exposure [14,15]. COPD is estimated to affect about 10% of the population over 45 years and to be the fourth cause of death worldwide. Unlike asthma, COPD is usually progressive and characterized by systemic inflammation and the development of lung fibrotic tissue. Additionally, due to the spread of corticosteroid resistance among patients and the lack of effective non-toxic medication, the need for new therapies is vital [14].

In this context, the purpose of the present work was to investigate the phytochemical profile, in terms of total polyphenols, flavonoids, and carotenoids, and in vitro radical scavenging, antioxidant, and antimicrobial activities of a bee-gathered polyfloral pollen extract (BPE). Moreover, the extracts’ anti-inflammatory effect on a tumor necrosis factor-alpha (TNF-α)-inflamed human pulmonary cell line (A549) was evaluated to assess bee pollen potential in mitigating pulmonary inflammation.

## 2. Materials and Methods

### 2.1. Chemicals and Reagents

All standards and reagents were of analytical grade. Folin–Ciocalteu (FC) reagent, sodium carbonate (Na_2_CO_3_), sodium acetate (C₂H₃NaO₂), potassium chloride (KCl), gallic acid, sodium hydroxide (NaOH), quercetin dihydrate, potassium hydrogen phosphate (K_2_HPO_4_), 6-hydroxy-2,5,7,8-tetramethylchromane-2-carboxylic acid (Trolox), potassium persulfate (K_2_S_2_O_8_), sodium nitrite (NaNO_2_), aluminum chloride (AlCl_3_), tris buffered saline with 1% Tween 20 (TBST), 2,2-diphenyl-1-picrylhydrazyl (DPPH), 2,4,6-Tri(2-pyridyl)-s-triazine (TPTZ), ferric chloride hexahydrate (FeCl_3_·6H_2_O), ferrous sulfate heptahydrate (FeSO_4_·7H_2_O), 2,2-azobis (2-amidinopropane) dihydrochloride (AAPH), fluorescein sodium salt, cell culture reagents, and medium supplements were purchased from Sigma-Aldrich (Spruce St, Saint Louis, MO, USA). Ethanol, methanol, acetone, and acetic acid were purchased from VWR (Radnor, PA, USA). Hydrochloric acid and the lyophilized culture medium Mueller–Hinton (MH) were purchased from Merck (Readington, NJ, USA).

### 2.2. Palynological Analysis and Bee Pollen Extraction

The Tuscan farm “Sapori Mediterranei” provided the organic bee pollen sample, harvested using beehives equipped with bottom-fitted pollen traps in San Rossore Regional Park in Lucca Province (Tuscany, Italy) from March to September 2018. After standard drying for commercialization, the sample was stored at room temperature until further analysis. Studio Naturalistico Il Pianeta Naturale (Valfabbrica, PG, Italy) performed the palynological analysis. The examination under the microscope was carried out at 1800× magnification. The relative frequencies of each pollen type were determined by counting at least 1000 pollen grains, following five parallel equidistant lines uniformly distributed over the entire observable field [16]. The Bee pollen sample was extracted and homogenized in 95% ethanol (BPE, 50 mg/mL) using an Ultraturrax (Kinematica Polytron PT MR 2100, Malters, Switzerland). After 1 h of incubation at room temperature with gentle stirring, the sample was centrifuged with a CR3 centrifuge (Jouan, Newport Pagnell, UK) for 10 min at 2700× *g* at 4 °C, and the supernatant was collected and kept in the dark at 4 °C until use. The extraction was carried out in triplicate.

### 2.3. Phytochemical Composition

#### 2.3.1. Total Polyphenols

The total polyphenols were assessed by measuring the reduction of the Folin–Ciocalteu (FC) reagent, as previously reported by Singleton et al. [17]. In brief, 100 µL of BPE was mixed with 500 µL of 0.2 N FC reagent and incubated in the dark for 5 min. Then, 400 µL of 0.7 M Na_2_CO_3_ was added, and the mixture was incubated for 2 h in the dark at room temperature. Absorbance was read at 760 nm with a FLUOstar Omega Microplate Reader (Software 5.7 R2, BMG LABTECH, Ortenberg, Germany), and results were expressed as mg of gallic acid equivalents (GAE) per g of fresh weight (mg GAE/g fw).

#### 2.3.2. Total Flavonoids

The determination of total flavonoids was performed with the aluminum chloride colorimetric reaction, adapting the method described by Kim et al. [18]. Shortly, 100 µL of BPE was mixed with 400 µL of H_2_O and 30 µL of 5% NaNO_2_. After 5 min of incubation at room temperature, 30 µL of 10% AlCl_3_ was added, and the mixture was incubated for 6 min. Finally, the reactions were stopped with 200 µL of 1 M NaOH and 240 µL of H_2_O. Absorbance was measured at 430 nm (FLUOstar Omega, BMG LABTECH, Ortenberg, Germany) after 30 min of incubation, and results were expressed as mg of quercetin equivalents (QE) per g of fresh weight (mg QE/g fw).

#### 2.3.3. Total Carotenoids

For carotenoids quantification, the method proposed by Kostić et al. [19] was followed. Bee pollen was extracted in 80% acetone (100 mg/mL) and homogenized (Ultraturrax, Kinematica Polytron PT MR 2100, Malters, Switzerland). The extraction was carried out overnight with constant shaking. Then, the samples were centrifuged for 10 min at 6620× *g* at 4 °C (Jouan CR31 centrifuge, Newport Pagnell, UK), and the supernatants were stored in the dark at 4 °C until use. The absorbance of 1 mL of extract was recorded at 450 nm (Perkin Elmer UV/VIS Lambda 365, Waltham, MA, USA), and results were expressed as µg of carotenoids over g of fresh weight (µg carotenoids/g fw).

### 2.4. In Vitro Antioxidant and Radical Scavenging Activities

#### 2.4.1. Ferric Reducing Antioxidant Power (FRAP) Assay

The ability of BPE to reduce ferric iron into ferrous ions was determined through the FRAP assay, with adaptations to the protocol previously described by Benzie and Strain [20]. In brief, FRAP buffer was prepared fresh by mixing 10 volumes of acetate buffer 300 mM (pH 3.6) with 1 volume of TPTZ 10 mM in HCl 40 mM, and 1 volume of FeCl_3_·6H_2_O 20 mM. A volume of 735 µL of FRAP buffer was added to 35 µL of BPE, and the mixture was kept in the dark for 30 min at room temperature. Absorbance was recorded at 593 nm (FLUOstar Omega, BMG LABTECH, Ortenberg, Germany), and results were expressed as mg (or µmol) of Fe^2+^ equivalents per g of fresh weight (mg or µmol Fe^2+^/g) using a FeSO_4_·7H_2_O (31.25–2000 µM) for the standard curve.

#### 2.4.2. DPPH Assay

According to Morais et al. [21], the BPE antiradical activity (ARA) towards the DPPH• radicals was assessed, with small modifications. Briefly, 25 µL of BPE was mixed with 975 µL of DPPH solution (60 µM in ethanol) and incubated for 30 min at 30 °C in the dark. Absorbance was recorded at 515 nm (FLUOstar Omega, BMG LABTECH, Ortenberg, Germany), and results were reported as EC_50_ (µg/mL), the concentration neutralizing 50% of DPPH• radical.

#### 2.4.3. Oxygen Radical Antioxidant Capacity (ORAC) Assay

The BPE’s ability to scavenge peroxyl radicals from the thermal degradation of AAPH was evaluated by ORAC assay, adapting the method of Ninfali et al. [22]. Shortly, 100 µL of BPE or 5 mM of the standard Trolox was mixed with 800 µL of the fluorescent probe fluorescein (40 nM in 0.075 M phosphate buffer, pH 7.4) and 100 µL of AAPH solution (400 mM in 0.075 M phosphate buffer, pH 7.4). The decay in fluorescence was recorded at λ_ex_ 485 nm and λ_em_ 514 nm (FLUOstar Omega, BMG LABTECH, Ortenberg, Germany), and results were expressed as µmol of Trolox equivalents (TE) per g of fresh weight (µmol TE/g fw).

### 2.5. Antimicrobial Activity

BPE activity towards the bacterial strains *Staphylococcus epidermidis* CNR-MLIP B206, *Bacillus subtilis* CNR-MLIP B038 (foodborne isolates maintained in the culture collection of the CNR-IBBA Microbiology Laboratory, CNR-MLIP), and *Pseudomonas stutzeri* DSM 5190 (clinical isolate from the DSMZ culture collection) was evaluated by the broth microdilution method (CLSI Standard M07-A10). BPE was filtered and then serially diluted in the MH culture broth. The bacterial strains were cultured in MH broth for 12 h at 30 °C and 120 rpm, and suspensions were adjusted to 1.5 × 10^4^ cells/mL for plate inoculation. For each well of a round-bottomed microwell plate, a volume of 50 µL of each sample dilution was mixed with 150 µL of microbial cells, to reach a final concentration of BPE ranging between 0.31 and 10 mg/mL. The MH culture broth (200 µL) was used as the blank reference, while the positive control of growth was represented by wells containing 50 µL of HM broth and 150 µL of microbial cell suspension for each bacterial strain. An additional microwell plate was prepared by mixing the relevant sample dilutions with 150 µL of sterile HM broth to obtain absorbance references for the different absorbances due to different sample concentrations. After 24 h incubation at 30 °C, the optical density (600 nm wavelength) was recorded using the FLUOstar Omega plate reader, and, after normalization using blank and extract absorbance data, cell growth was calculated based on a reference growth curve previously obtained from each of the selected strains.

### 2.6. A549 Cell Culture and Treatment

The human A549 cell line (ATCC^®^ CCL-185™; derived from lung alveolar epithelium) was cultured in high-glucose Dulbecco’s Modified Eagle Medium (DMEM) (Sigma-Aldrich Saint Louis, MO, USA). The medium was supplemented with 10% fetal bovine serum (FBS) and antibiotics (100 units/mL penicillin and 100 µg/mL streptomycin), and cells were maintained at 37 °C in a humidified environment with 5% CO_2_. For experiments, A549 cells were treated with DMEM without phenol red and FBS but still containing antibiotics. A549 cells were pre-incubated for 1 h with or without 10 or 50 µg/mL BPE, followed by 24 h exposure to 25 ng/mL TNF-α, or left untreated. Before determining the anti-inflammatory properties, cell viability was measured following 24 h exposure to increasing concentrations of BPE (0.5–200 µg/mL), using the MTT assay as previously described [23].

### 2.7. Gene Expression Analysis

RNA was extracted from A549 cells using the E.Z.N.A.^®^ Total RNA Kit I (OMEGA Bio-Tek, Norcross, GA, USA). The isolated RNA was then reverse transcribed into cDNA with the iScript™ cDNA Synthesis Kit (Bio-Rad, Hercules, CA, USA). Quantitative Real-Time PCR was carried out using the SsoFast™ EvaGreen^®^ Supermix (Bio-Rad, Hercules, CA, USA) on a CFX Connect Real-Time PCR Detection System (Bio-Rad, Hercules, CA, USA). The primers for the Interleukin-8 (IL-8), Cyclooxygenase-2 (COX2), and β-actin genes were as previously reported by Gabriele et al. [23]. Pre-developed PrimePCR SYBR Green Assay primers for Interleukin-1 beta (IL-1β, qHsaCID0022272) and Interleukin-18 (IL-18, qHsaCID0006163) were purchased from Bio-Rad (Hercules, CA, USA). Gene expression levels were determined using the 2^−ΔΔCT^ relative quantification method.

### 2.8. Immunoblot Analysis

For the Western blot analysis, total proteins from A549 cells were extracted for 30 min on ice using RIPA buffer supplemented with the cOmplete™ protease inhibitor cocktail (Sigma-Aldrich) and phosphatase inhibitor cocktail 3 (Sigma-Aldrich). The protein mixture was centrifuged at 12,000 xg for 30 min at 4 °C (Jouan CR 31 centrifuge; Newport Pagnell, UK), and the total content was determined via the Bradford assay (Sigma-Aldrich). A total of 45 μg of protein was separated by 10% SDS-PAGE and transferred to nitrocellulose membranes. The membranes were blocked with 5% non-fat milk for 2 h before being incubated overnight at 4 °C with specific primary antibodies against COX2, NF-kB, and GADPH (SAB5700721, SAB5700333, and SAB2108266, respectively; Sigma-Aldrich). Subsequently, the membranes were treated with peroxidase-conjugated secondary antibodies (A0545; Sigma-Aldrich) for 1 h at room temperature. After three washings with TBST, immunoreactive proteins were detected using a chemiluminescence kit (Clarity Western ECL substrates; Bio-Rad Laboratories, Hercules, CA, USA), and signals were captured using the ChemiDoc Imaging System (Bio-Rad Laboratories). The intensities of the protein bands were quantified using Image Lab Software version 6.1 (Bio-Rad Laboratories).

### 2.9. Statistical Analysis

Statistical analyses were conducted using GraphPad Prism version 9.00 for macOS (GraphPad Software, San Diego, CA, USA) and IBM SPSS Statistics v. 29.0.1.0 (IBM, NY, USA). All experiments were performed in triplicate, and the data are presented as mean ± standard deviation (SD). Differences between groups were evaluated using one-way analysis of variance (ANOVA), followed by Dunnett’s or Bonferroni’s post hoc tests for multiple comparisons. A *p*-value lower than 0.05 was considered statistically significant.

## 3. Results and Discussion

### 3.1. Palynological Analysis

The bee pollen studied here is a polyfloral sample composed of multiple botanical species, with none significantly dominating the composition. *Rubus ulmifolius* Schott was the most representative species (39.0%), followed by *Sambucus ebulus* L. (12.7%), *Quercus ilex* L. (10.6%), *Papaver rhoeas* L. (7.4%), *Lotus corniculatus* L. (5.9%), *Solidago virgaurea* L. (4.2%), *Coriandrum* sp. (3.0%), *Cistus* × *incanus* L. (2.5%), *Sambucus nigra* L. (1.7%), *Vicia faba* L. (1.6%), *Quercus robur* L. (1.5%), *Acer* sp. (1.4%), *Parthenocissus quinquefolia* (L.) Planch. (1.3%), and others (7.2%). Each pollen load had homogeneous and monospecific pollen content.

### 3.2. Phytochemical Composition Analysis

The presence of secondary metabolites in the BPE sample was evaluated based on their total polyphenol, flavonoid, and carotenoid content. Specialized metabolites are non-nutritive compounds biosynthesized by plants for defense, attraction, and hormonal functions. Among them, polyphenols represent a large and ubiquitous class, characterized by one or more phenol groups. Flavonoids, a common subclass with a peculiar three-ring structure, known as diphenylpropane, contribute to the color and flavor of foods [24]. Carotenoids are high-molecular-weight pigments with a tetraterpenoid structure. In addition to playing a crucial role in plants’ photosynthetic pathways, they give flowers and fruits their vibrant colors (yellow, orange, red, and purple) [25]. These and many other classes of compounds have been already detected in bee pollen samples [26].

In the BPE studied in the present work, the content of total polyphenols was found to be 20.2 ± 1.5 mg GAE/g fw, which is in line with the range of values previously reported for 10 polyfloral pollen samples harvested in Tuscany [7], as well as with polyfloral bee pollen from several sites of the Marche region (Italy) [27].

The BPE flavonoid and carotenoid contents were 9.22 ± 0.64 mg QE/g fw and 10.4 ± 1.4 µg/g fw, respectively. A similar quantity of carotenoids was previously found in a polyfloral bee pollen sample from Tuscany [12]. However, our results are substantially higher than those obtained by Kostić et al. [19] from a monofloral artichoke (*Cynara scolymus* L.) bee-gathered pollen, with amounts of phenolics, flavonoids, and carotenoids equal to 5.3 mg GAE/g dw, 0.81 mg QE/g dw, and 5.0 μg/g, respectively. Moreover, the flavonoids concentration in our sample fits in the range identified for a collection of monofloral and polyfloral bee pollens from Romania (4.93–20.45 mg QE/g), although the total phenolics (4.64–17.93 mg GAE/g) in our BPE are higher than theirs [28]. Additionally, phenolics and flavonoids resulted in higher levels in our sample compared to a collection of Moroccan mono- and polyfloral pollen loads [29].

Overall, a large variability in the phytochemical content of bee pollen samples can be appreciated in the literature. Indeed, as highlighted by several authors, this is the consequence of multiple factors, primarily the botanical origin of pollen grains, which reflects the unique biosynthetic pathways of each plant species, together with season and beehive activity [21,29,30].

### 3.3. Antioxidant and Antiradical Activities

The BPE’s antioxidant and radical scavenging potential was evaluated through the FRAP, DPPH, and ORAC assays. It is well known how phenolics- and carotenoid-rich natural products exert a protective effect towards pro-oxidative species, neutralizing them by giving away electrons or hydrogen atoms and resulting in innocuous products. Thus, for reliable results, it is advisable to evaluate the antioxidant activity through multiple assays, to probe every antioxidant mechanism [31].

In the FRAP assay, the extract antioxidant activity towards Fe^3+^ ions resulted in a value of 38.6 ± 4.5 mg Fe^2+^/g fw, corresponding to 139.01 ± 16.19 µmol Fe^2+^/g fw, which lies in the range previously identified for other polyfloral Tuscan bee pollens (14.77–190.27 µmol Fe^2+^/g fw) [7]. Additionally, BPE exerted a good radical scavenging ability against the DPPH• radical, with an EC_50_ of 613.8 ± 79.2 μg/mL, and towards the peroxyl radicals generated in the ORAC assay, with an activity of 433.77 ± 18.95 μmol TE/g fw. These results align with the published scientific literature. For instance, Castiglioni and colleagues [27] measured ORAC values oscillating from 300.1 to 801.6 µmol TE/g dw in their collection of polyfloral pollens from the Marche region. Additionally, 28 mono- and polyfloral Chilean honeybee pollens displayed ORAC values between 109 and 492 µmol TE/g fw [32]. Regarding the DPPH assay, our EC_50_ value is lower but comparable to those recorded for a chestnut bee pollen sample from Anatolia (0.857 ± 0.060 mg/mL) and much lower than those detected in a collection of mono- and polyfloral pollens from five Portuguese natural parks (2.16–5.87 mg/mL) [21,33]. However, the polyfloral Moroccan pollen sample studied by Bakour et al. [34] exerted a superior radical scavenging activity towards the DPPH• radicals (50.35 ± 2.27 μg/mL) and Fe^3+^ reduction activity in the FRAP assay (208.73 ± 2.04 µmol Fe^2+^/g). Again, the remarkable differences in antioxidant assays are attributable to the variable chemical compositions of the samples mentioned above.

### 3.4. Antimicrobial Activity Analysis

The BPE was screened for its growth-inhibitory activity against Gram-positive foodborne bacterial strains of *Staphylococcus epidermidis* and *Bacillus subtilis*, and against a Gram-negative clinical isolate of *Pseudomonas stutzeri* through the broth microdilution assay. *S. epidermidis*, a skin-resident bacterium, and *P. stutzeri*, commonly found in soil and other environmental matrices, are known for causing human infections, especially in clinical settings [35,36]. Along with *B. subtilis*, a soil-derived bacterium capable of forming resistant spores, strains belonging to the selected species may be responsible for foodborne diseases and/or food spoilage [37]. These species may represent bacterial models characterized by different metabolic abilities and environmental growth strategies, useful in studies assessing the antimicrobial activity of compounds intended for human consumption.

**Figure 1:**
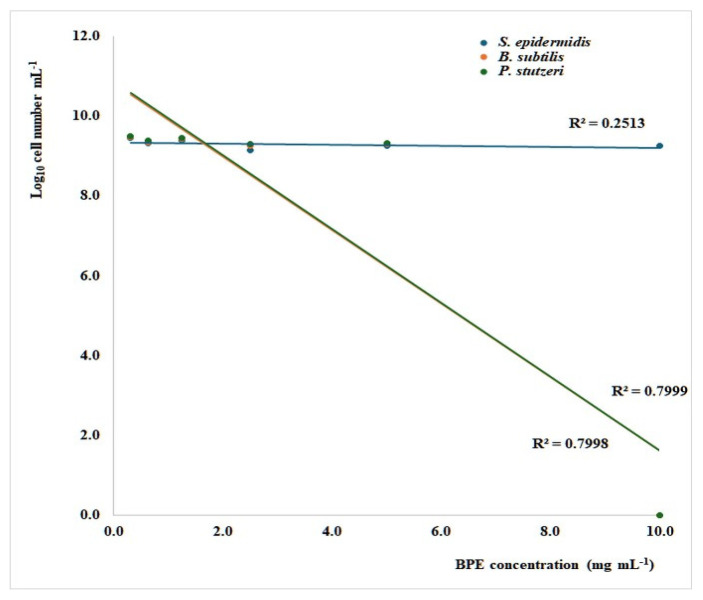
Linear regression curves indicating the relationship between the growth of *Staphylococcus aureus*, *Bacillus subtilis*, and *Pseudomonas stutzeri* after 24 h of culture in microdilution broth assays and the concentration of bee pollen extract (BPE). The significance of regression is 0.023 (*S. aureus*) and <0.001 (*B. subtilis* and *P. stutzeri*).

Both *P. stutzeri* and *B. subtilis* were susceptible to BPE, with a minimum inhibitory concentration (MIC) of 10 mg/mL (Figure 1), which is consistent with our previous work, where the MIC for *Cistus*, *Castanea*, and *Rubus* bee pollen extracts were found to range between 5 and 10 mg/mL for a selection of pathogenic Gram-positive and -negative bacteria [38]. Inhibiting concentrations between 2.5 and 5 mg/mL for Gram-positive bacteria and 5 to 10 mg/mL for Gram-negative ones were also reported for an ethanolic polyfloral bee pollen extract [39]. In the present investigation, the BPE did not arrest *S. epidermidis* growth at the tested concentrations, showing a maximum growth reduction of 33%, compared with controls at 10 mg/mL (Figure 1).

Nevertheless, a very wide range of behaviors, from no sensitivity to very low MIC values, was reported for the different strains of this species [33,39,40,41]. Interestingly, when testing low concentrations of Anatolian bee pollen extracts from *Castanea sativa* for their growth-inhibitory activity, Sonmez et al. [33] noticed that the ethanolic extract was effective only against *Mycobacterium smegmatis* (MIC < 15.62 µg/mL), while the methanolic extract was significantly more potent, being effective towards 14 Gram-positive and -negative strains, with MIC values between 62.5 and 500 µg/mL. The methanolic extracts were also effective in an investigation comparing the antimicrobial potential of eight monofloral and polyfloral commercial bee pollens, although the activity towards pathogenic bacteria varied depending on the pollen sample and the bacterial strain, with MIC ranging from 1.81 to 9.42 mg/mL [42]. All this evidence suggests that bee pollen is an antimicrobial agent active towards a broad spectrum of microorganisms; on the contrary, the extent of its activity depends on its origin and chemical composition.

### 3.5. Investigation of the Anti-Inflammatory Effect of BPE in Inflamed A549 Cells

The bioactivity of BPE was evaluated in A549 lung cells (type II pneumocytes) under inflammatory stress. Airway tissue inflammation is involved in various respiratory conditions, including the common COPD, where a range of inflammatory cells and mediators are activated by oxidative stress caused by exposure to cigarette smoke or other toxicants [43]. Since bee pollen extract is full of specialized metabolites, such as phenolics and carotenoids, which exhibit excellent antioxidative properties and have shown benefits for chronic diseases, it is important to analyze its potential therapeutic effects further.

**Figure 2:**
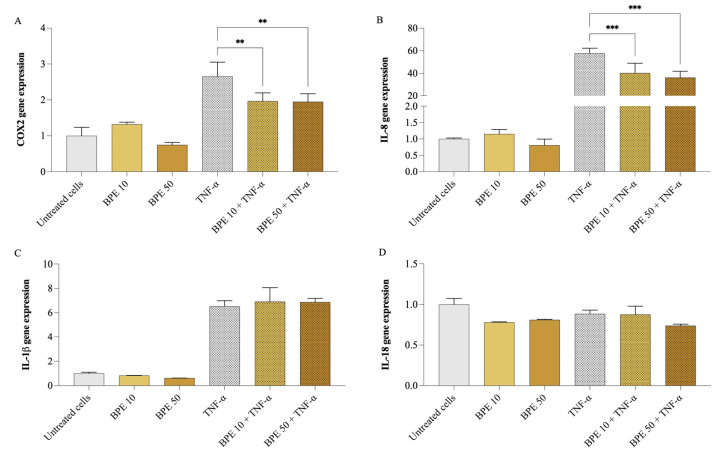
Impact of bee pollen extract on the expression of genes linked to inflammation. COX2 (**A**), IL-8 (**B**), IL-18 (**C**), and IL-1β (**D**) gene expression. A549 cells were pretreated for 1 h with 10 µg/mL (BPE 10) or 50 µg/mL (BPE 50) bee pollen extract, then exposed 24 h with 25 ng/mL TNF-α. One-way ANOVA and Dunnett’s post hoc test: * different from TNF-α, ** *p* < 0.01, *** *p* < 0.001.

Firstly, cell viability was assessed using the MTT assay to ensure that BPE did not exert cytotoxic effects. None of the BPE concentrations up to 50 µg/mL (200 µg/mL maximum concentration tested) negatively affected A549 cell viability. Subsequently, A549 cells were pretreated for 1 h with 10 and 50 µg/mL of BPE, followed by exposure (or not) to 25 ng/mL of TNF-α for 24 h. As shown in Figure 2, exposure of A549 cells to 25 ng/mL of TNF-α resulted in a significant upregulation of inflammatory mediator gene expression, particularly, IL-8 (*p* < 0.001) increased 57 times, COX2 (*p* < 0.001) doubled, and IL-1β (*p* < 0.001) was 6 folds higher, compared to untreated cells. However, no change was observed in IL-18 gene expression. Pretreatment with BPE at both concentrations significantly reduced the IL-8 and COX2 expression, while no significant effect was observed on IL-1β expression. Precisely, BPE 50 caused COX2 expression to go down by 26.7%, while that of IL-8 by 37.2%.

**Figure 3:**
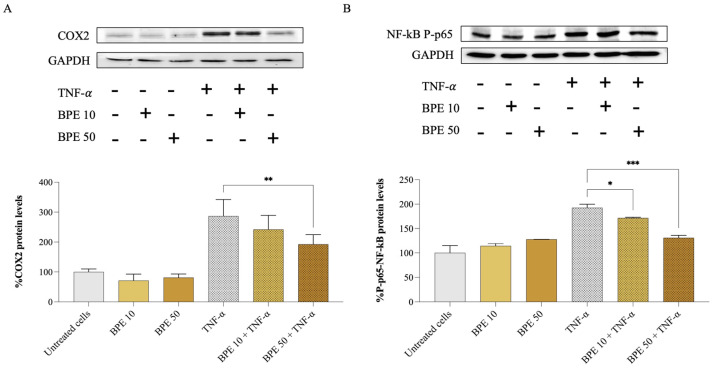
Effect of bee pollen extract on inflammation-related protein levels. Western blot analysis and semi-quantitative results of COX2 (**A**) and phospho–NF-κB p65 (**B**). GAPDH was used as a loading control. A549 cells were pre-incubated for 1 h with either 10 (BPE 10) or 50 (BPE 50) µg/mL bee pollen extract and exposed for 24 h to 25 ng/mL TNF-α. Data were analyzed using one-way ANOVA and Dunnett’s post hoc test: * different from TNF-α, * *p* < 0.05, ** *p* < 0.01, *** *p* < 0.001.

At the protein level, the effect of BPE on A549 inflamed cells was confirmed by monitoring the expression of COX2 and phospho–NF-κB p65 proteins by Western blotting. Compared to the control, TNF-α-inflamed cells showed a significant 3-fold increase in COX2 (Figure 3A) and 2-fold increase in phospho–NF-κB p65 (Figure 3B) proteins. However, the treatment with BPE 50 downregulated COX2 protein levels by 32.9% in TNF-α-stimulated A549 cells. According to the study by Lopes and colleagues [44], a bee pollen hydroalcoholic extract could also inhibit the activity of COX1 and COX2 isolated enzymes by 86 and 91%, respectively, at 50 µg/mL concentration.

Furthermore, our analysis revealed that BPE affects the activation of the NF-κB signaling pathway. We found that both BPE 10 and 50 reduced the phosphorylation of the NF-κB p65 subunit (Figure 3B), a key step in the activation of this nuclear factor, by 10.9% and 31.8%, respectively. Bee pollen’s ability to interfere with the NF-κB signaling cascade could potentially explain the reduced expression of pro-inflammatory genes observed in A549 cells exposed to TNF-α. Notably, NF-κB activation has been observed in COPD patients, where it induces the transcription of genes encoding cytokines and chemokines (e.g., IL-1β, IL-8, and TNF-α), promoting the migration of inflammatory cells and contributing to further inflammation and disease progression [43].

Overall, research on the protective effects of honeybee pollen against lung inflammation and injury is limited. To the best of our knowledge, this is the first study assessing the anti-inflammatory activity of bee pollen on A549 lung cells subjected to inflammatory stress. In the ChaGo-K-1 lung cancer cell line, the exposure to bee pollen protein hydrolysates (molecular weight < 0.65 kDa) induced apoptosis at a 1.37 µg/mL concentration [45]. One available in vivo study examined the anti-inflammatory effects of polyphenol-rich bee pollen from *Camellia sinensis* L. in a lipopolysaccharide (LPS)-induced acute lung injury mouse model [46]. In this study, the oral administration of 300 mg/kg of the pollen extract for 7 days, followed by a 1 mg/kg LPS injection, reduced the infiltration of inflammatory cells in lung tissue. Moreover, immunohistochemistry revealed that the pollen extract more than halved the expression of COX2 and inflammasome NLRP3 in lung cells, performing better than dexamethasone, which was used as the gold standard [46]. Additionally, a polyfloral bee pollen phenolic extract and its isolated flavonoid component, myricetin, showed anti-allergic effects in a mouse model of ovalbumin-induced allergy [47]. A dose of 200 mg/kg of such extract reduced paw edema size by 54%, decreased the levels of anaphylactic IgE and IgG1 (−29% and −27%), and attenuated leukocyte migration (−52%) in the lung tissue. Similarly, myricetin (5 mg/kg) brought down the leukocyte count (−43%) and the number of eosinophils (−73%) [47]. Moreover, our recent study demonstrated that hemp seeds and sprouts also exhibit anti-inflammatory effects on A549 lung cells, further highlighting the potential of plant-based bioactive compounds in combating inflammation-related respiratory diseases [48].

Encouraging results in both cell- and animal-based experiments have also been obtained from testing bee pollen in other oxidative and inflammation-related conditions. For instance, in the study performed by Li Liang et al. [49], bee pollen from *Camellia sinensis* L. was tested on an inflammatory bowel disease model using Caco-2 cells exposed to dextran sulfate sodium. Pretreatment with the polyphenol-rich extract reduced the cytotoxic effects of dextran sulfate, upregulated antioxidant mRNAs, and downregulated inflammatory ones. Furthermore, in a steatosis model on Hepa1–6 cells, polyfloral bee pollen treatment reduced by half lipid accumulation and improved AAPH-induced oxidative stress [32]. Lotus bee pollen (100–500 μg/mL) was shown to ameliorate an isoproterenol-induced cardiomyocyte injury in rat H9c2 cells by increasing the activity of antioxidant enzymes while reducing the expression of pro-inflammatory and pro-apoptosis mediators in a dose-dependent manner [50]. Instead, in LPS-stressed RAW 264.7 mouse macrophages, treatment with monofloral bee pollen extracts from *Quercus acutissima* Carr. and *Actinidia arguta* Planch. significantly reduced the release of pro-inflammatory and pro-oxidative mediators [51]. In particular, bee pollen from *A. arguta* was more effective, inducing a reduction in prostaglandin E2 and nitric oxide production by 66.23 ± 0.1% and 78.21 ± 0.06%, respectively, together with a d 60 ± 0.9% decrease in ROS release. Concurrently, the extracts modulated the mitogen-activated protein kinase (MAPK) pathway, while stimulating nuclear factor erythroid 2-related factor 2 (Nrf2) and decreasing NF-κB p65 nuclear translocation [51]. Additionally, polyfloral bee pollen (250 mg/kg) reduced neuroinflammation in a hamster model of autism, normalizing the increased levels of IL-6 (327.03%) and the decrease in IL-10 (−64.11%) [52]. Moreover, in rats subjected to chronic immobilization stress, bee pollen (200 mg/kg) exerted beneficial effects by increasing brain-derived neurotrophic factor in the hippocampus while lowering inflammatory mediator levels (IL-1β and TNF-α) in brain tissue [53].

Other natural extracts and phenolic compounds have similarly been shown to reduce inflammation in inflamed A549 cells via several pathways, including modulation of NF-κB, nicotinamide adenine dinucleotide phosphate (NADPH) oxidase 2, and MAPK, as well as upregulation of sirtuin-1 [54,55,56]. Thus, it is not surprising that honeybee pollen exhibits similar effects.

A final consideration should be addressed concerning bee pollen, that is, its allergic potential. Although allergic reactions are mostly caused by wind-pollinated species, some individuals might still be sensitive to bee-gathered pollen due to the presence of allergenic proteins [57,58]. Therefore, this aspect must not be neglected when assessing the nutraceutical value of this superfood.

## 4. Conclusions

In the present research work, we gathered additional information on the nutraceutical properties of bee pollen, which confirms itself as an excellent natural product due to its desirable phytochemical composition, and antioxidative and antimicrobial activities. Furthermore, we demonstrated its efficacy in mitigating inflammation induced in A549 airway epithelial cells, suggesting its utility in preventing various diseases associated with this process, including COPD. These features, combined with the not-so-expensive nature of bee pollen, make it a suitable candidate for further study and pharmaceutical applications.

## Data Availability

The original contributions presented in this study are included in the article. Further inquiries can be directed to the corresponding author.
